# NFC-Based Wearable Optoelectronics Working with Smartphone Application for Untact Healthcare

**DOI:** 10.3390/s21030878

**Published:** 2021-01-28

**Authors:** Min Hyung Kang, Gil Ju Lee, Joo Ho Yun, Young Min Song

**Affiliations:** 1 School of Electrical Engineering and Computer Science, Gwangju Institute of Science and Technology, 123 Cheomdangwagi-ro, Buk-gu, Gwangju 61005, Korea; kminh9409@gist.ac.kr (M.H.K.); gjlee0414@gist.ac.kr (G.J.L.); dvswnghrnt@gmail.com (J.H.Y.); 2 Anti-Virus Research Center, Gwangju Institute of Science and Technology, 123 Cheomdangwagi-ro, Buk-gu, Gwangju 61005, Korea; 3 AI Graduate School, Gwangju Institute of Science and Technology, 123 Cheomdangwagi-ro, Buk-gu, Gwangju 61005, Korea

**Keywords:** untact healthcare, wearable healthcare, NFC based, smartphone application, compact

## Abstract

With growing interest in healthcare, wearable healthcare devices have been developed and researched. In particular, near-field communication (NFC) based wearable devices have been actively studied for device miniaturization. Herein, this article proposes a low-cost and convenient healthcare system, which can monitor heart rate and temperature using a wireless/battery-free sensor and the customized smartphone application. The authors designed and fabricated a customized healthcare device based on the NFC system, and developed a smartphone application for real-time data acquisition and processing. In order to achieve compact size without performance degradation, a dual-layered layout is applied to the device. The authors demonstrate that the device can operate as attached on various body sites such as wrist, fingertip, temple, and neck due to outstanding flexibility of device and adhesive strength between the device and the skin. In addition, the data processing flow and processing result are presented for offering heart rate and skin temperature. Therefore, this work provides an affordable and practical pathway for the popularization of wireless wearable healthcare system. Moreover, the proposed platform can easily delivery the measured health information to experts for contactless/personal health consultation.

## 1. Introduction

Peoples interest in well-being is increasing with rising concerns about their health such as pandemic [1,2], aging [3], and chronic diseases [4,5]. However, with the growing interest, the increase in medical expenses has affected as an economic burden for both individuals and governments [5,6,7,8]. Accordingly, wearable healthcare devices have been researched and developed with various bio-signal sensing (e.g., physical [9,10,11], thermal [12,13,14], and cardiovascular [15,16,17,18] signals). Among them, the thermal and cardiovascular signals can be an important indicator for diagnosing patient conditions [19]. In particular, heart rate and skin temperature are useful for daily health checking, symptom monitoring for infection as well [19,20]. Recently reported wearable healthcare devices have mainly focused on device miniaturization and wireless operation (i.e., Bluetooth and near-field communication (NFC)) for user convenience [21,22,23]. Although the wearable device has mainly adopted Bluetooth system, because of the built-in battery, the disadvantage of its bulky size and heavyweight can degrade the user’s comfort [23]. Thus, recently, NFC-based devices have been proposed to enhance body comfort owing to its advantages of battery-free and wireless [21,23]. Due to such simple and compact features, various applications based on NFC have been reported, such as colorimetric sensing of sweat [24], “skin-like” device monitoring heart rate variability (HRV) [25], epidermal ultra-violet dosimeter [26], smart-contact lens [27], miniaturized pulse oximetry [28], and wireless electronic tattoo [18]. The principle of NFC is an electromagnetic induction that occurs between two coil-type inductors, and the induced power can be maximized by matching the resonance frequency of the two coils, which are included in the NFC device and NFC reader, respectively [23,29]. For stronger magnetic power induction, higher inductance is required, which is predominantly determined by the number of turns [29]. Since larger number of turns needs larger dimension, device miniaturization, and high magnetic power have a trade-off relation. Meanwhile, the NFC reader not only acts as a power supply to the NFC device, but can also read data from the NFC device [23,29]. In this context, NFC-enabled smartphone can also provide power to the NFC device [23,29]. However, for data communication between smartphone and NFC device, smartphone application must be developed. Moreover, the smartphone application can improve convenience for wearable healthcare by combining with mobile healthcare system, providing personalized health consultation from experts (i.e., doctors, nurses, dietitians, health trainers, and so on) through mobile devices such as smartphone and tablet PC [30,31].

Herein, this study suggests a convenient healthcare platform, which is a compact and inexpensive system using a wireless/battery-free sensor compatible with a smartphone. The wireless device is engineered based on the NFC system and adopts a dual-layer layout for the efficiency of power transfer. Three-dimensional ray-tracing simulation computationally studies the encapsulation layer effect on the accurate data acquisition. Additionally, to achieve wireless/battery-free operation, the electrical optimization process of NFC-based circuit is performed, and the smartphone application is programmed. Based on these optical and electrical optimizations, the device is implemented by the conventional semiconductor processing and simple soldering step. The flexibility of the fabricated device is demonstrated by showing the device operation in bent state and cyclic bending test. In addition, the adhesive strength is confirmed between the device and the skin. Due to this flexibility and adhesion, the device can be firmly attached and operate at various body sites such as wrist, fingertip, temple, and neck, while continuously communicating with a smartphone. Finally, the accurate heart rate and temperature are obtained by performing the post-processing.

## 2. Result and Discussion

**Figure 1:**
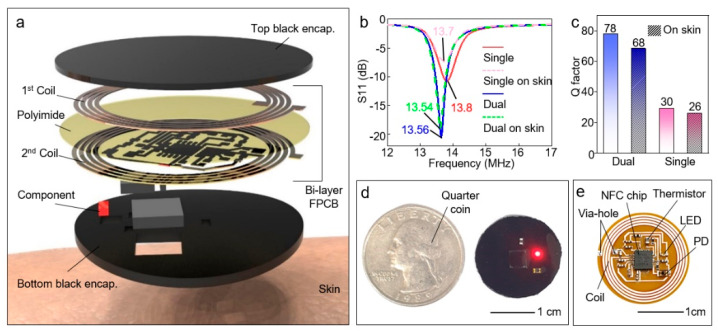
Near-field communication (NFC) based wireless device. (**a**) Exploded view of schematic illustration of the wireless device. (**b**) Measured frequency characteristics of single-layer and dual-layer devices in both cases of devices on a slide glass and devices on skin. (**c**) Q-factor of dual-layer and single-layer device attached to the skin. (**d**) Image of black encapsulated device with Quarter coin. (**e**) Photograph of unencapsulated wireless device.

Figure 1a displays the exploded view of wireless sensor to show constitution of each layer. The wireless device composed of dual-layer coil, circuit, electric components (i.e., NFC chip, light-emitting diode (LED), photodiode (PD), thermistor, resistors, and capacitors) and black encapsulation layer. For stronger magnetic power with a smaller size, a dual-layer coil structure is adopted to achieve a greater number of turns in the same dimension. The dual-layer coil is separated by a polyimide layer and connected through a via-hole. Furthermore, resonance frequency (*f*_res_) matching was performed to enable communication with the NFC reader. As displayed in Figure 1b, the dual-layer device has strong attenuation and sharp resonance curve than the single-layer device. Accordingly, Q-factor of the dual-layer device is higher than the single-layer device (Figure 1c). The S11 data of the device was measured by a vector network analyzer (VNA) (E5071C ENA, Keysight, Santa Rosa, CA, USA) and RF near-field probe (PBS1, AARONIA, Strickscheid, Germany). Since the dual-layer coil can achieve more turns in the same dimension compared with single layer coil, and thus more inductive magnetic power can be generated. The equation relative between inductance and number of turns is as follows:

(1) $$L_{c}=\frac{N^{2}\mu A}{l}$$

where *L*_c_ is the inductance of the coil, *N* is the number of turns in wire coil, *μ* is permeability of material, and *A* is area of coil. The Q-factor is determined by *L*_c_, *C*_c_, and the coil resistance (*R*_c_). The S11 magnitude mode was set to measure the *f*_res_ and Q-factor of the coil. The *f*_res_ of the coil is the frequency resulting in minimum reflected power. The *f*_res_ equation and Q-factor equation were used to design the coil, which can be expressed as

(2) $$\text{Q-factor}=\frac{1}{R_{c}}\sqrt{\frac{L_{c}}{C_{c}}}\approx \frac{f_{\mathrm{res}}}{f_{2}-f_{1}}$$

(3) $$f_{\mathrm{res}}=\frac{1}{2\pi \sqrt{L_{c}C_{c}}}$$

where *L*_c_ is the inductance of the coil and *C*_c_ is the total capacitance of the device. The Q-factor is determined by *L*_c_, *C*_c_, and the coil resistance (*R*_c_). The Q-factor represents the sharpness of the resonance peak, and thus can be defined as an approximate calculation, where *f*_2_ and *f*_1_ is −3 dB frequency of *f*_res_ and *f*_2_ − *f*_1_ is the bandwidth of *f*_res_.

In addition, as demonstrated in Figure 1b,c, the frequency characteristics of our device are barely changed even in contact with skin unlikely other NFC devices [18,32]. Normally, frequency characteristics are degraded due to the substantial capacitive loading induced by skin, which is body loading effect [33,34]. We minimized the body loading effect by optimizing the NFC coil and device feature. Futher, because of its small size, deformation caused by attaching to the body barely affects the device performance. Therefore, our device can operate attached on skin without performance degradation. In the NFC chip datasheet, the supplied and rectified coil power is typically 2V, and the data rate is 6–26 kbps [35]. However, in order to achieve these values, optimized frequency characteristics is important. The test board designed and fabricated by an NFC chip manufacturer has a Q-factor of 67.8 [36]. Since the Q-factor of our device is 68 even in attached to the skin, our device can sufficiently achieve the efficiency of power and data rate specified on the NFC datasheet. Therefore, we optimized the device structure to maximize the performance of the NFC chip.

As shown in the Figure 1d, the device is encapsulated by black elastomer (the mixture of black dye (~10 wt%) and Q1-4010, Dow Corning Corporation, Midland, MI, USA; elastomer for printed circuit board) to block the direct transmission of light from the LED to the PD. In addition, the black encapsulation layer can protect a circuit from degradation and contact with skin. Thus, leakage current flowing to skin can be blocked. Moreover, the current value for driving LED measured by parameter analyzer (4156C, Agilent, Santa Clara, CA, USA) is ~1.66 mA, which is not harmful to humans [37]. In the safety data sheet of polymer for the black encapsulation layer, material safety is verified for humans [38]. Furthermore, a biocompatible double-sided tape (PC2723U, ScapaHealthcare, Marlborough, UK), which has been utilized in other wearable devices [28], is used for adhesion with skin. Therefore, the safety of our devices is verified in terms of electrical and material perspective. The quarter coin highlights the compact size of the device. As exhibited in Figure 1e, the wireless device includes the NFC chip (RF430FRL152H, Texas Instruments, Dallas, TX, USA) and coil for wireless operation, which enable operate LED (APTD1608SEC, Kingbright, New Taipei City, Taiwan), and read detected data from PD (VEMD1060X01, Vishay, Malvern, PA, USA) and thermistor (NCU18WF104D6SRB, Murata, Nagaokakyo, Japan) for biological data acquisition (Figure 1b).

**Figure 2:**
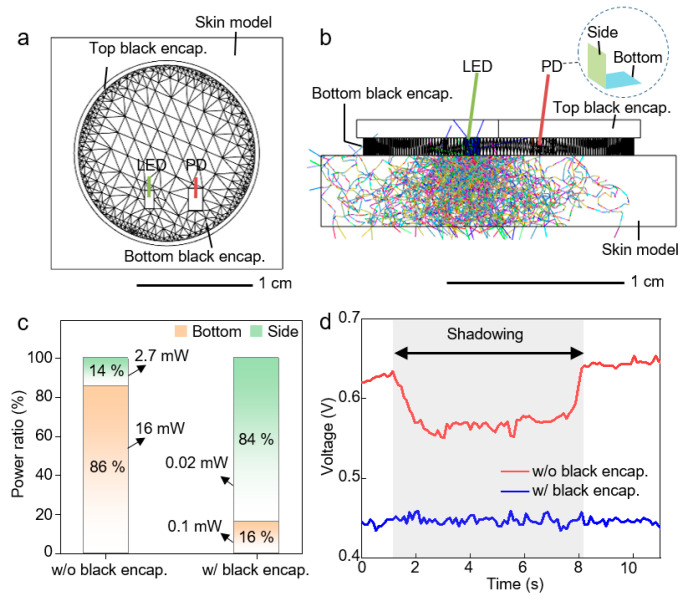
Investigation on the optical interference to the device. (**a**) Top image of simulation structure. (**b**) Side view of a simulation structure with 10,000 rays applied. (**c**) Detected light power at side and bottom of photodiode (PD) in both simulations with and without black encapsulation. (**d**) Measured light signal as applying shadow in both cases of the black encapsulated device and bare device.

Since all circuit metal lines of our device are encapsulated, electrical interference can be blocked. Preferably, an optical interference can affect the performance of device. In order to block internal and external optical noise, black encapsulation was applied to the top and bottom of the device. Ray-tracing simulation was performed to confirm blocking optical noise by black encapsulation. As displayed in Figure 2, the simulation structure was designed with similar features to the device. In addition, to consider the absorption and scattering in the skin, optical properties of skin were applied to the skin model. Figure 2b shows that black encapsulation can block the direct light from LED to PD without passing through the skin. As a result of simulation in Figure 2c, since the black encapsulation layer can block the direct light from LED to PD, collected light power at side of PD is lower than the light power at bottom. However, in case of no black encapsulation, light power at side of PD is higher than the power at bottom. Moreover, the collected total light power is much higher when not including black encapsulation. Therefore, the black encapsulation layer can screen the external optical noise. Finally, by comparing the measured light signal with and without black encapsulation, we confirmed that the black encapsulated device was hardly affected by optical interference (Figure 2d). The gray area noted by shadowing indicates that an opaque object shades the ambient light.

**Figure 3:**
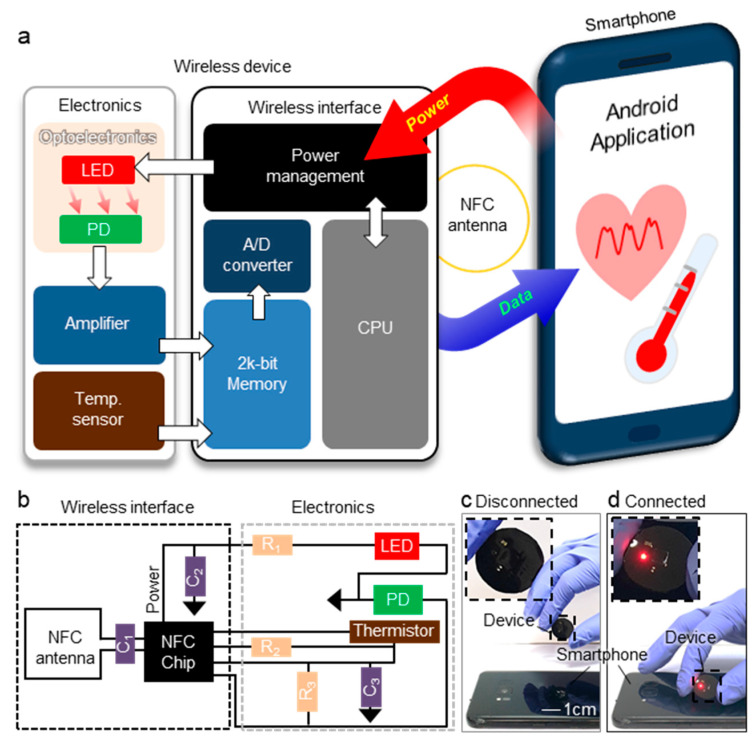
Operating system of wireless sensor. (**a**) Block diagram for operation of wireless sensor. (**b**) Circuit diagram of wireless sensor. Photograph images of device in state of (**c**) disconnected and (**d**) connected with smartphone.

The device operation is engineered based on NFC system. As shown in the block diagram for device operation (Figure 3a), the operating system is divided into three parts: the first is the NFC reader (i.e., smartphone, tablet, etc.) that supplies power to the device and obtain data transferred from the wireless device. The second part is the wireless interface composed of NFC chip and coil for wireless communication with smartphone. The third is electronics parts with a LED, PD, and thermistor for bio-measurements. Figure 3b explains circuit diagram of device. R_2_ (100 kΩ) is reference resistor for thermistor. R_3_ (5 MΩ) is amplification resistor. C_1_ (9 pF) is resonance capacitor for resonance frequency tuning of NFC system. C_2_ (0.1 μF) and C_3_ (1 μF) are decoupling capacitor to remove noise. R_1_ (1.1 kΩ) is LED resistor. The circuit is designed by referring to the NFC chip datasheet, which includes a detailed circuit diagram for operation of the NFC chip [35]. The detailed circuit diagram is shown in Supplementary Figure S1. As demonstrated in Figure 3c,d, the device can operate wirelessly without battery connected with smartphone. After the smartphone supplies power to the device, the NFC chip of device rectifies the power and supplies to the LED to emit light. The PD detects the backscattered light from the blood vessel. The thermistor and reference resistor provide raw data corresponding to temperature. The device transfers the collected data to the smartphone wirelessly.

Table 1 compares our device with commercial sensors in terms of the operating system, power supply, biological signals, weight, and size. Our proposed device not only deals with wireless/battery-free operating system but also provides heart rate and temperature with the lightest weight and the smallest size.

**Figure 4:**
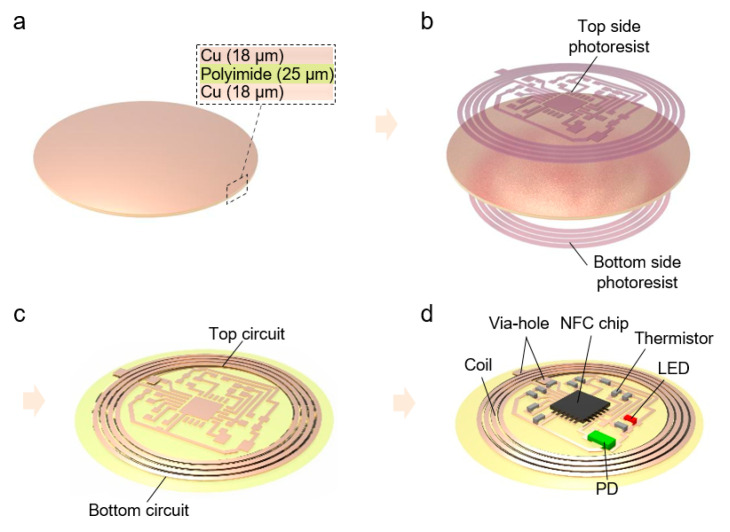
Fabrication process. (**a**) Preparation for Cu/PI/Cu foil. (**b**) Double-sided photolithography for pattering. (**c**) After Cu wet etching with etchant. (**d**) Soldering electric component soldering and forming via-hole with silver paste.

Figure 4 exhibits fabrication process of the device. A Cu (18 μm)/PI (25 μm)/Cu (18 μm) foil (SME, Korea) was used to fabricate dual-layered FPCB (Figure 4a). The photolithography process on each side of foil was performed with photoresist (PR, AZ9260, AZ Electronic Materials, Luxembourg) and developer (AZ 300 MIF, AZ Electronic Materials, Luxembourg) (Figure 4b). The double-side patterned sample is etched with Cu etchant (CE100, TRANSENE, Danvers, MA, USA) (Figure 3c). Finally, the electrical components were soldered on the fabricated FPCB with silver paste (BST-506, BEST, China) (Figure 4d). Moreover, drilling holes formed the via-holes using a micro drill bit, and the filled silver paste offers the electrical path between top and bottom coils. Therefore, the dual-layer device can be achieved through the overall process.

**Figure 5:**
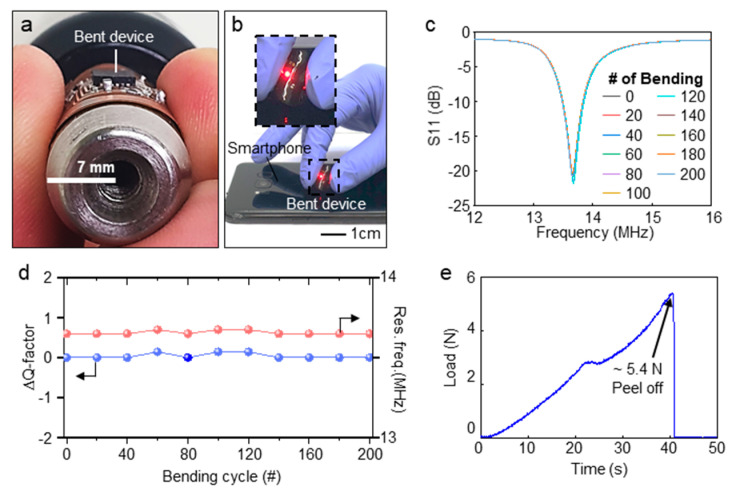
Frequency characteristics and bending test for wireless sensor. (**a**) Photograph image of bent device with RoC of 7 mm. (**b**) Picture of operating device in bent state. (**c**) S11 data of device measured by vector network analyzer (VNA) with cyclic bending stress. (**d**) Variation in Q-factor and resonance frequency of device in cyclic bending stress. (**e**) Peeling of test for evaluation device adhesion with skin.

The frequency characteristics of device is measured with bending stress (Figure 5a–c). As shown in Figure 5a, the device has high flexibility with ~7 mm of Radius of Curvature (RoC), considering that the RoC of the finger is 13 mm which is the smallest RoC among typical measurement site [45,46,47]. Furthremore, the device can operate connected with smartphone even in bent state (Figure 5b). A cyclic bending test is performed to confirm the durability of the device against bending stress. For the test, the device was bent 200 times with a cylindrical object as displayed in Figure 5a. As exhibited in Figure 5c, the S11 data of the device maintain the almost same value during the repetitive bendings. In addition, as presented in Figure 5d, frequency features are verified in terms of Q-factor and resonance frequency (Res. Freq.) which are barely changed during the cyclic bending test. A biocompatible double-sided tape is used for adhesion with skin. Figure 5e exhibits the result of adhesion test of the device with skin using a peeling-off tester (text analyzer pulse, Micro stable, UK) on the device mounted on the skin. The measured adhesion strength is suitable for devices attaching to the skin compared with reported devices [28,48].

**Figure 6:**
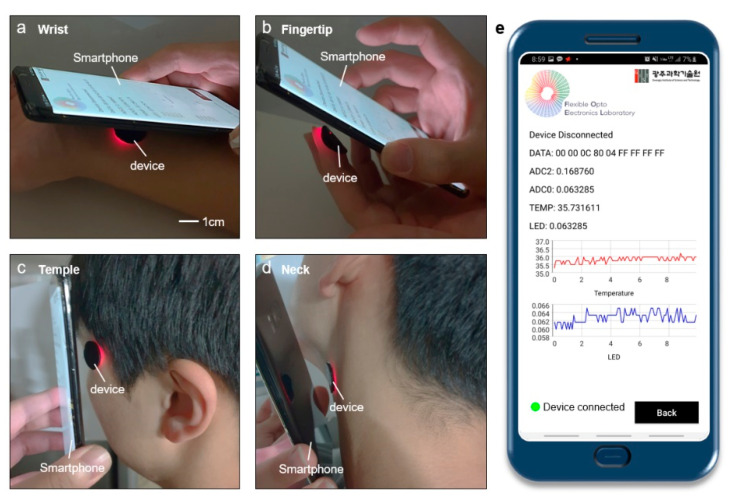
Measurement at various sites such as (**a**) fingertip, (**b**) wrist, (**c**) temple, (**d**) neck. (**e**) Captured image of the smartphone application of smartphone displaying data measured at wrist.

As exhibited in Figure 6a–d, based on the distinguished flexibility and adhesion, the device can be attached to various body sites. However, in order to obtain an accurate heart rate from the blood, the pulse signal should be measured at a limited area of the body where arterial blood flows from not deep into the skin, such as the wrist, fingertips, temple, forehead, and neck [46,47]. Figure 6e displays the screen of the smartphone application while measuring the biological signals at the wrist. The smartphone application is developed by commercial software (Android Studio, Google, Mountain View, CA, USA) based on JAVA program language. The bio-signals detected by PD and thermistor are collected to smartphone by using the smartphone application. The smartphone application is programmed to send commands to the wireless device for operation and communication. The obtained data from PD and thermistor are plotted in real-time and saved in smartphone with 10 Hz sampling rate. The communication distance between the device and smartphone is maximally ~1 cm for ensuring stable operation. Therefore, the device can attach on various areas of body, and the smartphone can collect measured data by using the customized application.

**Figure 7:**
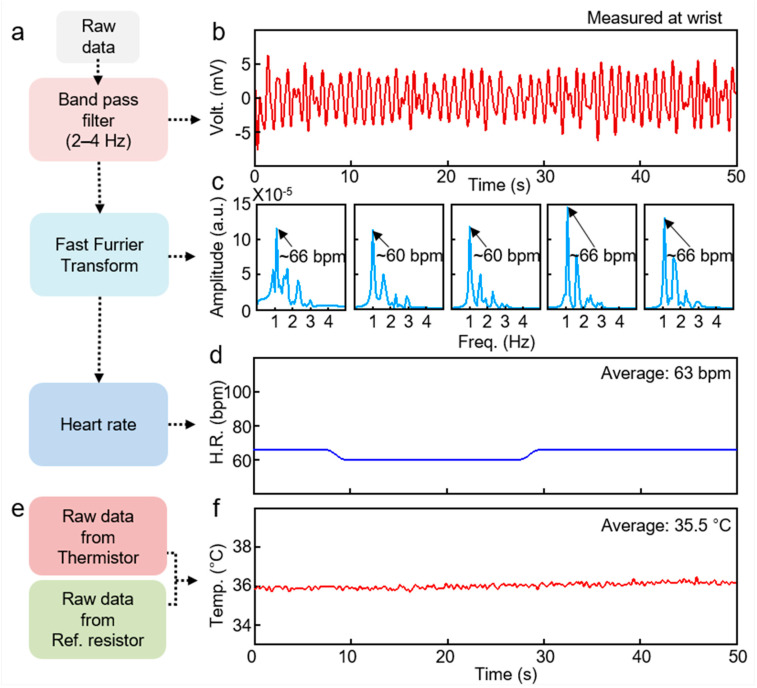
Flow chart and results for data processing. (**a**) Data processing flow for heart rate. (**b**) Pulsatile signal obtained by PD. (**c**) Fast Furrier Transform (FFT) result of the signal in (**b**) with 10-s window. (**d**) Heart rate calculated from (**c**). (**e**) Data processing flow for temperature. (**f**) Measured temperature.

Figure 7 shows the data processing flow and results for heart rate and skin temperature. To obtain heart rate, the raw data from PD is processed (Figure 7a–d). First, a bandpass filter was applied to the raw data to eliminate noise for clear pulsatile signals. Variation in blood flow during systolic and diastolic of cardiac causes the pulsatile signals, which is a periodic signal from the heartbeat [33,34]. Second, Fast Furrier Transform (FFT) is applied to the pulsatile signals with a 10-s window to acquire the frequency value of the heartbeat [17]. By multiplying the frequency value by 60, the heart rate per minute can be obtained. The temperature calculation is based on the variation in raw data from thermistor compared with reference resistor (Figure 7e). The varied resistance of the thermistor depending on the temperature generates a difference in voltage applied to the thermistor and the reference resistor. The detailed equations to calculate the temperature value are referred to the NFC chip datasheet [35]. Therefore, the post-processing method and processed results are presented for acquiring heart rate and temperature.

Table 2 shows the average heart rate and skin temperature and standard deviations from two subjects. A normal resting heart rate for adults ranges from 60 to 100 beats per minute. Therefore, all the subjects are normal state. This study is our initial step about untact healthcare, and the exhaustive study will be conducted by recruiting more subjects in subsequent papers. In many previously reported papers, experiments were conducted in this form [17,49].

## 3. Conclusions

In this work, we propose an affordable and practical untact healthcare system, which composed of a wireless/battery-free sensor and customized smartphone application. The wireless device can provide heart rate and skin temperature for health monitoring. We applied a dual-layered coil to the device for stronger induced magnetic power with compact size. In addition, we optimized and fabricated NFC based circuit, which includes the circuit for detection of heart rate and skin temperature. The smartphone application was developed to collect and real-time plot the measured data from the wireless device. We experimentally demonstrated that the device can operate as it attached to various body sites such as wrist, fingertip, temple, and neck owing to exceptional flexibility and adhesion. In addition, accurate heart rate and temperature were obtained through post-processing of raw data. Based on the experimental verification and methods, we believe that this study can provide insight and useful information for the popularization of wireless healthcare system. In future work, we will conduct exhaustive study by recruiting more subjects.

## 4. Experimental Section

Device Fabrication method: The Cu/PI/Cu foil coated with PR by spin coater. The coated sample with PR was exposure to ultra-violet light for 15 s. In order to prevent damage on patterned PR during the process on the opposite side, hard-bake is performed to the one-side patterned sample with a temperature of 120 °C for 5 min. The patterned sample developed for 2 min with developer. Wet etching for the sample is performed for 8min. The electrical components are soldered at temperature of ~200 °C with hot plate.

In Vivo Experiment: Before proceeding with the experiment, the subject was allowed to acclimate to the environment for approximately 120 s, sitting comfortably. The device was attached to the wrist, finger, temple and neck. The bio-signals were captured by an NFC reader at a distance of ~1 cm. All of the in vivo experiments in this study were performed in compliance with the protocol approved by the institutional review board at Gwangju Institute of Science and Technology (GIST). A healthy subject, aged 20–30 years, participated in the study. Informed consent was obtained from subject involved in the study.

## Figures and Tables

**Table 1:** Specifications of commercial wearable sensors in terms of operating system, power supply, biological signals, weight, and size.

Product	Operating System	Power Supply	Biological Signals	Weight	Size (mm^3^)
This work	Wireless (NFC)	Battery-free	Heart rate, temperature	1 g	20 × 20 × 2
[39]	Wireless (Bluetooth)	Battery	Temperature	3 g	64 × 35 × 4.5
[40]	Wireless (Bluetooth)	Battery	Temperature	45 g	99 × 25 × 48
[41]	Wireless (Bluetooth)	Battery	Heart rate	60 g	200 × 80 × 3
[42]	Wireless (Bluetooth)	Battery	Temperature	15 g	43 × 43 × 16
[43]	Wireless (Bluetooth)	Battery	Temperature	3 g	28 × 26 × 3.5
[44]	Wireless (Bluetooth)	Battery	Heart rate	18 g	100 × 23.1 × 8.3

**Table 2:** Mean of calculated heartrate and skin temperature for subjects.

	Mean Heart Rate (bpm)	STD	Mean Skin Temp. (°C)	STD
Subject 1	63	2.7	35.5	0.18
Subject 2	64	2.96	35.1	0.19

## Data Availability

Data sharing not applicable.

## Supplementary Materials

The following are available online at https://www.mdpi.com/1424-8220/21/3/878/s1, Figure S1: Circuit diagram of designed device. The dashed areas in NFC chip indicate the pin positions. The empty pin positions of NFC chip are electrically open states. The detailed information related to pin position is described in data sheet. The distance between LED and PD was set to 4 mm.
