# Supplementary material for: NFC-Based Wearable Optoelectronics Working with Smartphone Application for Untact Healthcare
Source: Sensors (Basel). 2021 Jan 28;21(3):878. doi: 10.3390/s21030878 (PMC7865650; doi:10.3390/s21030878)
Supplement: Supplementary file 1 [file sensors-21-00878-s001.pdf]

Supplementary information for

NFC-based Wearable Optoelectronics Working with Smartphone Application for Untact Healthcare

Min Hyung Kang [†,1], Gil Ju Lee [†,1], Joo Ho Yun [1] and Young Min Song [*,1,2,3]

[1]School of Electrical Engineering and Computer Science, Gwangju Institute of Science and Technology, 123 Cheomdangwagi-ro, Buk-gu, Gwangju 61005, Korea; kminh9409@gist.ac.kr (M.H.K.); gjlee0414@gist.ac.kr (G.J.L.); dvswnghrnt@gmail.com (J.H.Y.)
[2]Anti-Virus Research Center, Gwangju Institute of Science and Technology, 123 Cheomdangwagi-ro, Buk-gu, Gwangju 61005, Korea
[3]AI Graduate School, Gwangju Institute of Science and Technology, 123 Cheomdangwagi-ro, Buk-gu, Gwangju 61005, Korea

* Correspondence: ymsong@gist.ac.kr; Tel.: +82-62-715-2658
† These authors contributed equally to this work.

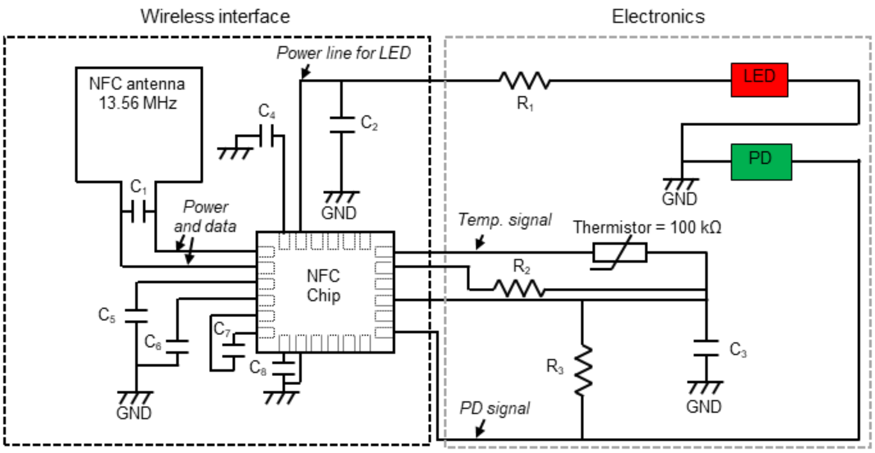

Element	Value
C_1	9 pF
C_2	0.1 μF
C_3	1 μF
C_4	1 μF
C_5	2.2 μF
C_6	0.1 μF
C_7	10 nF
C_8	0.1 μF
R_1	1.1 kΩ
R_2	100 kΩ
R_3	5 MΩ

Figure S1. Circuit diagram of designed device. The dashed areas in NFC chip indicate the pin positions. The empty pin positions of NFC chip are electrically open states. The detailed inform ation related to pin position is described in data sheet. The distance between LED and PD was set to 4 mm.
